# Supplementary material for: Transcription factor binding sites are frequently under accelerated evolution in primates
Source: Nat Commun. 2023 Feb 11;14:783. doi: 10.1038/s41467-023-36421-3 (PMC9922303; doi:10.1038/s41467-023-36421-3)
Supplement: Supplementary file 1 — Supplementary Information [file 41467_2023_36421_MOESM1_ESM.pdf]

Supplementary material for:

Transcription factor binding sites are frequently under accelerated evolution in primates

Xinru Zhang[1,2,3*], Bohao Fang[4] and Yi-Fei Huang[1,2*]

[1]Department of Biology, Pennsylvania State University,

University Park, PA 16802, USA

[2]Huck Institutes of the Life Sciences, Pennsylvania State University,

University Park, PA 16802, USA

[3]Bioinformatics and Genomics Graduate Program, Pennsylvania State University,

University Park, PA 16802, USA

[4]Department of Organismic and Evolutionary Biology and the Museum of Comparative Zoology, Harvard University, Boston, MA 02135, USA

*Correspondence: yuh371@psu.edu and xmz5176@psu.edu

Supplementary Table 1: Non-overlapping TFBS groups under accelerated evolution. r_1 and r_2 are the relative substitution rates of a TFBS group in the human lineage and in other primates. P-values are calculated from likelihood ratio test. The ratio (r_1/r_2) indicates the fold of increase in substitution rate in the human lineage.

Genomic elements	r_1	r_2	*P*-value	r_1/r_2
Pol III binding	2.30	1.41	0	1.63
BDP1	1.34	1.12	1.41E-06	1.19
POU5F1-NANOG binding	0.91	0.83	0.002	1.10
POU5F1	1.03	0.94	1.5E-05	1.09
FOXP2	0.99	0.92	0	1.08
NANOG	0.90	0.85	0.004	1.05
NRF1	1.16	1.11	0.03	1.05

Supplementary Table 2: Relative BICs for different foreground lineages.

Model	Foreground lineage	Pol III binding	BDP1	POU5F1-NANOG binding	POU5F1	FOXP2	NANOG	NRF1
M1	Human	158.29	23.27	9.82	18.74	141.34	8.24	4.79
M2	Hominini	530.38	35.65	24.47	36.96	199.91	16.67	30.24
M3	Homininae	1060.78	40.06	24.11	23.77	211.00	10.36	33.54
M4	Hominidae	1590.02	65.20	15.94	17.96	320.93	20.07	46.08
M5	Caterrhini	1973.45	113.72	8.81	36.88	186.56	13.15	47.28
M6	Simiiformes	1110.76	58.78	2.09	20.54	28.66	0.13	6.30
M7	Haplorhini	0	0	0	0	0	0	0

Supplementary Table 3: Rates of adaptive substitutions estimated by the INSIGHT model. D_p indicates the expected number of divergences per kilobase driven by positive selection in the human lineage. SE$[D_p]$ is the standard error of D_p. P-values are calculated from the one-sided Wald test.

Genomic elements	D_p	SE$[D_p]$	*P*-value
HARs	7.67	0.14	< 0.0001
Pol III binding	7.28	0.59	< 0.0001
BDP1	0.60	0.35	0.40
POU5F1-NANOG binding	0	0	
POU5F1	0	0	
FOXP2	0	0	
NANOG	0	0	
NRF1	0.27	0.26	0.15

Supplementary Table 4: Estimates and confidence Intervals from the mixture model.

Genomic elements	$1-\hat{\pi}_{ub}$	95%CI of $1-\pi_{ub}$	$\hat{a}$	95%CI of a	$\hat{\lambda}$	95%CI of λ
Pol III binding	0.78	(0.74, 0.81)	0.22	(0.19, 0.26)	8.0E-6	(3.06E-7, 3.42E-5)
BDP1	0.20	(0.18, 0.24)	0.30	(0.22, 0.37)	0.71	(0.71, 0.72)
POU5F1-NANOG binding	0.08	(0.04, 0.11)	0.43	(0.30, 0.66)	0.86	(0.65, 0.94)
POU5F1	0.10	(0.07, 0.15)	0.38	(0.35, 0.47)	0.83	(0.71, 0.89)
FOXP2	0.27	(0.25, 0.29)	0.52	(0.51, 0.54)	0.43	(0.37, 0.49)
NANOG	0.26	(0.23, 0.29)	0.55	(0.47, 0.55)	0.42	(0.37, 0.56)
NRF1	0.25	(0.22, 0.29)	0.45	(0.41, 0.52)	0.54	(0.38, 0.54)

Supplementary Table 5: Group-level LRT estimates on ChIP-seq peaks of histone modifications. r_1 and r_2 are the relative substitution rates of a collection of ChIP-seq peaks in the human lineage and in other primates. The ratio (r_1/r_2) indicates the fold of increase in substitution rate in the human lineage. *P*-values are calculated from two-sided likelihood ratio test.

Species	Regions	Tissue	Fold of increase in substitution rate(r_1/r_2)	*P*-value
Rhesus macaque	promoter	liver	0.95	0
Rhesus macaque	enhancer	liver	1.00	0.04
Human	promoter	liver	0.92	0
Human	enhancer	liver	0.98	0
Rhesus macaque	CRE	brain	0.97	0
Chimpanzee	CRE	brain	1.04	0
Human	CRE	brain	1.05	0
