# Supplementary material for: Transcription factor binding sites are frequently under accelerated evolution in primates
Source: Nat Commun. 2023 Feb 11;14:783. doi: 10.1038/s41467-023-36421-3 (PMC9922303; doi:10.1038/s41467-023-36421-3)
Supplement: Supplementary file 3 — Description of Additional Supplementary Files [file 41467_2023_36421_MOESM3_ESM.pdf]

Description of Additional Supplementary Files

File Name: Supplementary Data 1
Description: Group-level LRT results of 161 groups of binding sites. r_1 and r_2 are the relative substitution rates of a TFBS group in the human lineage and in other primates. N indicates the length of concatenated sequence of a TFBS group. P-values (p) are calculated from chi-square test of likelihood ratio. Bonferroni-corrected p-values are presented in the column named Adj.p. The ratio (r_1/ r_2) indicates the fold of increase in substitution rate in the human lineage.

File Name: Supplementary Data 2
Description: Significant GO biological process terms of accelerated TFBS associated genes. Sheet 1 contains GO terms of the genes associated with top accelerated TFBSs in all the seven groups. Sheet 2 contains GO terms of the genes associated with top accelerated TFBSs in FOXP2 group.

File Name: Supplementary Data 3
Description: Group-level LRT results of CTCF tissue-specific binding sites. Acession ID and tissue information are listed. r_1 and r_2 are the relative substitution rates of a tissue-specific CTCF binding site group in the human lineage and in other primates. The ratio (r_1/ r_2) indicates the fold of increase in the substitution rate of the CTCF
