# Supplementary material for: Transcription factor binding sites are frequently under accelerated evolution in primates
Source: Nat Commun. 2023 Feb 11;14:783. doi: 10.1038/s41467-023-36421-3 (PMC9922303; doi:10.1038/s41467-023-36421-3)
Supplement: Supplementary file 7 — Reporting Summary [file 41467_2023_36421_MOESM7_ESM.pdf]

Corresponding author(s): Yi-Fei Huang; Xinru Zhang

Last updated by author(s): 01/18/2023

Reporting Summary

Nature Portfolio wishes to improve the reproducibility of the work that we publish. This form provides structure for consistency and transparency in reporting. For further information on Nature Portfolio policies, see our Editorial Policies and the Editorial Policy Checklist.

Statistics

For all statistical analyses, confirm that the following items are present in the figure legend, table legend, main text, or Methods section.

n/a	Confirmed	
☐	☒	The exact sample size (*n*) for each experimental group/condition, given as a discrete number and unit of measurement
☐	☒	A statement on whether measurements were taken from distinct samples or whether the same sample was measured repeatedly
☐	☒	The statistical test(s) used AND whether they are one- or two-sided<br>*Only common tests should be described solely by name; describe more complex techniques in the Methods section.*
☐	☒	A description of all covariates tested
☐	☒	A description of any assumptions or corrections, such as tests of normality and adjustment for multiple comparisons
☐	☒	A full description of the statistical parameters including central tendency (e.g. means) or other basic estimates (e.g. regression coefficient) AND variation (e.g. standard deviation) or associated estimates of uncertainty (e.g. confidence intervals)
☐	☒	For null hypothesis testing, the test statistic (e.g. *F*, *t*, *r*) with confidence intervals, effect sizes, degrees of freedom and *P* value noted<br>*Give P values as exact values whenever suitable.*
☒	☐	For Bayesian analysis, information on the choice of priors and Markov chain Monte Carlo settings
☒	☐	For hierarchical and complex designs, identification of the appropriate level for tests and full reporting of outcomes
☐	☒	Estimates of effect sizes (e.g. Cohen's *d*, Pearson's *r*), indicating how they were calculated

Our web collection on statistics for biologists contains articles on many of the points above.

Software and code

Policy information about availability of computer code

Data collection	No software was used in data collection. Data were downloaded from publicly available resources (See the "Data" section for details).
Data analysis	Statistical analysis were carried out using bedtools (v2.27.1), R v4.0.3, ape (v5.5) package, phangorn (v2.7.1) package, ClassComparison(3.1.8) package, stringr (v1.4.0) package, rphast v1.6.9 package. The scripts for GroupAcc are available on https://github.com/May-BG/GroupAcc/tree/v1.0.0 (Doi: 10.5281/zenodo.7535878). Selection analysis script available on https://github.com/CshlSiepelLab/FitCons2.

For manuscripts utilizing custom algorithms or software that are central to the research but not yet described in published literature, software must be made available to editors and reviewers. We strongly encourage code deposition in a community repository (e.g. GitHub). See the Nature Portfolio guidelines for submitting code & software for further information.

Data

Policy information about availability of data

All manuscripts must include a data availability statement. This statement should provide the following information, where applicable:

- Accession codes, unique identifiers, or web links for publicly available datasets
- A description of any restrictions on data availability
- For clinical datasets or third party data, please ensure that the statement adheres to our policy

Human TFBS annotation were downloaded from Txn Factor ChIP Track on UCSC genome browser. Primate alignments were extracted from Multi alignment of 46 vertebrate genomes from the UCSC Genome Browser (http://hgdownload.cse.ucsc.edu/goldenPath/hg19/multiz46way/). Reference model built with concatenated

alignments of all TFBSs was uploaded to https://github.com/May-BG/GroupAcc/tree/v1.0.0 (Doi: 10.5281/zenodo.7535878). TFBS groups with accelerated evolution in primates were uploaded to the GitHub page. Previously defined HARs collections were downloaded from https://docpollard.org/research/. ChIP-seq data of primates' brains were downloaded from NCBI GEO Series GSE67978 and Accession E-MTAB-2633. Human tissue-specific CTCF binding site information were downloaded from ENCODE with the accession numbers from supplementary data 3.

Human research participants

Policy information about studies involving human research participants and Sex and Gender in Research.

Reporting on sex and gender	N/A
Population characteristics	N/A
Recruitment	N/A
Ethics oversight	N/A

Note that full information on the approval of the study protocol must also be provided in the manuscript.

Field-specific reporting

Please select the one below that is the best fit for your research. If you are not sure, read the appropriate sections before making your selection.

☐ Life sciences ☐ Behavioural & social sciences ☒ Ecological, evolutionary & environmental sciences

For a reference copy of the document with all sections, see nature.com/documents/nr-reporting-summary-flat.pdf

Ecological, evolutionary & environmental sciences study design

All studies must disclose on these points even when the disclosure is negative.

Study description	We developed two pooling based methods (GroupAcc) to study both strongly and weakly accelerated evolution in human and primates. We grouped the binding sites by the transcription factors that could bind to them. In the first method, we concatenated primate alignments for each binding site in a group and then applied likelihood-ratio test to the concatenated alignments. In the second method, we first applied likelihood-ratio test to the alignments of each binding site, then calculated the empirical p-values from parametric bootstrapping, and then fit a beta-uniform mixture model to the distribution of p-values.
Research sample	The ten-species alignments were extracted from the Multiz alignment of 46 vertebrate genomes from the UCSC Genome Browser. In order to study accelerated evolution, especially weakly accelerated evolution in regulatory elements, we included the following samples: TFBS groups found in the human genome annotation of ENCODE Txn Factor ChIP tracks on UCSC Genome Browser; previously defined HARs collection as positive control downloaded from https://docpollard.org/research; H3K4me3 enriched regions and H3K27ac enriched regions in liver of 20 mammals including human and rhesus macaque (Accession E-MTAB-2633); H3K27ac enriched regions in human, chimpanzee and rhesus macaque brain from NCBI GEO Series GSE67978, and CTCF tissue-specific binding sites found on ENCODE.
Sampling strategy	There are 161 TFBS groups from ENCODE track and each group contains thousands of binding sites with 100-300 bp long. We included all the 161 TFBS groups without predetermining the sample size because simulation showed that GroupAcc can estimate the accelerated evolution on the concatenated alignments with stronger statistical power.
Data collection	The ten-sequence alignments were extracted from the Multiz alignment of 46 vertebrate genomes from the UCSC Genome Browser. Samples include: TFBS groups found in the human genome annotation of ENCODE Txn Factor ChIP tracks on UCSC Genome Browser; previously defined HARs collection as positive control downloaded from https://docpollard.org/research; H3K4me3 enriched regions and H3K27ac enriched regions in liver of 20 mammals including human and rhesus macaque (Accession E-MTAB-2633); H3K27ac enriched regions in human, chimpanzee and rhesus macaque brain from NCBI GEO Series GSE67978, and CTCF tissue-specific binding sites found on ENCODE.
Timing and spatial scale	The source code of ENCODE was available in Nov 2018.
Data exclusions	We extracted the alignments of TFBSs across ten primate species using rphast. We removed the TFBSs overlapping with UTRs, CDSs and previously identified HARs. To filter out low-quality alignments, we obtained informative alignment sites where unambiguous bases were found in at least five out of ten primate species in the Multiz alignment. We retained TFBSs with at least 50 informative sites for downstream analysis.
Reproducibility	This study did not include any laboratory or field experiments. Results of the analyses based on empirical data were reported. All the original data were downloaded from open-source platforms like UCSC genome browser and ENCODE. Scripts for the analyses is available on the Github page and can be replicated by others.
Randomization	The empirical p-values for the element-level LRTs were estimated from parametric bootstrapping. We also generated synthetic

Randomization	alignments based on the reference model under various evolutionary dynamics. The GroupAcc methods performed well in the synthetic data in various scenarios.
Blinding	NA.

Did the study involve field work? ☐ Yes ☒ No

Reporting for specific materials, systems and methods

We require information from authors about some types of materials, experimental systems and methods used in many studies. Here, indicate whether each material, system or method listed is relevant to your study. If you are not sure if a list item applies to your research, read the appropriate section before selecting a response.

Materials & experimental systems

n/a	Involved in the study
☒	☐ Antibodies
☒	☐ Eukaryotic cell lines
☒	☐ Palaeontology and archaeology
☒	☐ Animals and other organisms
☒	☐ Clinical data
☒	☐ Dual use research of concern

Methods

n/a	Involved in the study
☒	☐ ChIP-seq
☒	☐ Flow cytometry
☒	☐ MRI-based neuroimaging
